# Direct Feeding at the Breast Is Associated with Breast Milk Feeding Duration among Preterm Infants

**DOI:** 10.3390/nu9111202

**Published:** 2017-11-01

**Authors:** Shiran Pinchevski-Kadir, Shir Shust-Barequet, Michal Zajicek, Mira Leibovich, Tzipi Strauss, Leah Leibovitch, Iris Morag

**Affiliations:** 1 The Edmong and Lily Safra Children Hospital, Chaim Sheba Medical Center, Sackler School of Medicine, Tel Aviv University, Ramat Gan 5262000, Israel; shiranpinch@gmail.com (S.P.-K.); t.tzipi@gmail.com (T.S.); leah.leibovitch@gmail.com (L.L.); 2 Rappoport Faculty of Medicine, Technion—Israel Institute of Technology, Haifa 3525422, Israel; shirshust@gmail.com; 3 Department of Obstetrics and Gynecology, Chaim Sheba Medical Center, Tel Hashomer, Sackler Faculty of Medicine, Tel Aviv University, Ramat Gan 5262000, Israel; mic.zajicek@gmail.com; 4 Newborn Neonatal Unit, Mayanei Hayeshua Medical Center, Bnei-Brak 5154475, Israel; drmleibovich@gmail.com

**Keywords:** breast milk, exclusive breast milk feeding, direct feeding at the breast, expressed breast milk, preterm infants

## Abstract

Background: In spite of high rates of initiating breast milk feeding (BMF) among preterm infants, a significant rate of discontinuation occurs shortly after discharge. Aim: To investigate the effect of mode (direct feeding at the breast vs. expressing) and exclusivity (breast milk combined with formula vs. breast milk only) as well as maternal perceptions on the duration of BMF among preterm infants. Methods: The study included mothers whose infants were born before 32 weeks gestation, between January 2012 and August 2015 at Sheba Medical Center (SMC). Perinatal data were collected retrospectively from infants’ computerized charts. Mothers were approached >12 months postpartum and were asked to complete a questionnaire. Those who agreed to participate were asked (during their visit to the follow-up clinic or by phone or mail) to complete a questionnaire regarding mode and duration of BMF as well as reasons for its discontinuation. Mothers were also asked about their pre-partum intentions to feed directly at the breast. Results: Out of 162 eligible mothers, 131 (80.8%) initiated BMF during their intensive care unit (ICU) hospitalization. Of these, 66 (50.3%) discontinued BMF earlier than six months postpartum. BMF ≥ 6 months was significantly associated with direct feeding at the breast, duration of exclusive BMF, and singleton birth. Regression analysis revealed that direct feeding at the breast (any or only) and duration of BMF exclusivity were the only significant variables associated with BMF duration (Odds ratio (OR) 5.5 and 95% confidence interval (CI) 2.00–15.37; OR 1.5 and 95% CI 1.25–1.88, respectively). Milk supply (inadequate or nonexistent) was the most commonly reported cause for BMF discontinuation <6 months. Direct feeding at the breast was significantly associated with BMF duration and was more common among singletons. Conclusions: Direct feeding at the breast and duration of exclusive BMF are associated with duration of BMF among infants born <32 weeks of gestational age (GA). These findings suggest that targeting these two factors may play a key role in prolonging BMF duration among preterm infants.

## 1. Background

It is well known that breast milk feeding during the first months of life is extremely beneficial. Studies have shown that breast milk feeding (BMF) among preterm infants results in better short- and long-term outcomes [1,2,3]. The WHO and the American Academy of Pediatrics (AAP) recommend “exclusive breastfeeding for about 6 months, followed by continued breastfeeding as complementary foods are introduced, with continuation of breastfeeding for 1 year or longer as mutually desired by mother and infant” [4,5,6]. Despite the above recommendations, the incidence and duration of BMF among mothers of preterm infants continue to be lower than among mothers of full-term infants [7]. Breast milk feeding of preterm infants presents unique challenges that include establishing milk production, maintaining a milk supply and later transitioning from gavage feeding to direct feeding at the breast [7]. Multiple factors have been shown to be associated with BMF initiation and continuation, among them maternal demographics reflecting basic maternal condition (age, education, income, marital status, ethnicity, other children in the family, employment, and duration of maternity leave), infant characteristics and medical status (gestational age, birth weight, gender, multiple infant births, medical condition, and length of hospitalization) [8,9,10], mode of feeding (expressed milk by bottle or direct at the breast), timing of initiation of BMF and exclusivity of BMF [11,12,13,14], maternal perception of successful milk production and transfer to the infant [15,16], the hospital and neonatal intensive care unit (NICU) mentality [14,17,18], and interventions to promote educate and support mothers regarding BMF [14,19,20,21,22,23].

In our recent study, we found that infants whose mothers failed to supply ≥75% of daily nutrition as BMF at Day 14 of life were more likely to be fed exclusively by formula six weeks after discharge [24].

In the present study, we attempt to expand our understanding by further investigating the effect of BMF mode and exclusivity on BMF duration.

## 2. Methods

The study included mothers whose infants were born before 32 weeks of gestation, between January 2012 and August 2015 at Sheba Medical Center (SMC). Mothers were asked to participate in the study if they were >12 months postpartum. Exclusion criteria included death (maternal of infant) prior to discharge from the NICU, major congenital malformations or known genetic disorders, and triplet pregnancies (as they reflect a different population with respect to maternal challenges in starting and continuing BMF). Perinatal data were collected retrospectively from infants’ computerized charts. Those who agreed to participate were asked by one of 3 of the investigators (I.M., L.L., or S.P.-K.) to complete a questionnaire regarding mode and duration of BMF as well as reasons for its discontinuation (see Supplementary Material). Mothers could choose to complete the questionnaire during their visit to the follow-up clinic or by phone or mail. If a question was asked, the interviewer was available to answer further questions.

The computerized system of the SMC neonatal department (MetaVision, iMD soft, Dusseldorf, Germany) served as a platform for organizing the data, which included maternal demographics (age, years of education, income, marital status) and obstetric characteristics (fertility treatment, gestational age, and delivery mode).

Our department protocol prioritizes BMF. Donor breast milk is not yet available in Israel. Mothers are strongly encouraged and guided by the medical team and lactation consultants to express their own milk for their babies soon after birth. “An individualized developmental care program carried out in the NICU includes early skin-to-skin contact as well as parental education for active care of infants from admission through discharge. Although parents may enter the NICU and remain beside their infant thought the day (except during nurses’ shift changes), there are no single rooms or other facilities for rooming in. The space is divided into four large halls, with 14–24 isolettes or cribs in each. Privacy can be attained using portable dividers” [24].

This program is directed by a developmental team comprising a physiotherapist, an occupational therapist and a physician who is a NIDCAP (neonatal individualized care and assessment program) professional. Moreover, lactation consultants and a speech therapist guide support the parents on a daily basis from admission until discharge. Direct feeding at the breast is commenced when the infant reaches 33 weeks. Infants are discharged home at 36 weeks gestational age (GA) if they meet the following criteria: having a weight of >1.9 kg and a stable temperature, being free of apneic episodes, and exhibiting mature oral feeding with adequate growth.

For the purpose of this study, we determined the following definitions for type of nutrition: (i) Breast milk feeding (BMF)—infants whose nutrition included breast milk feeding of any kind, regardless of whether direct feeding at the breast- or bottle-feeding (equal to breast milk feeding strictly superior to 0%); (ii) Exclusive BMF (EBMF)—infants who were fed maternal breast milk exclusively and did not receive any formula (equal to 100% breast milk feeding).

The following definitions pertain to feeding technique (regardless of whether exclusive or combined with formula): (iii) Direct feeding at the breast—infants who were fed directly at the breast whether combined with bottle-feeding (any) or not (only); (iv) Expressed breast milk (bottle-feeding)—infants whose nutrition included breast milk that was exclusively expressed and given via bottle. We used the term BMF rather than breastfeeding as mothers of preterm infants have to express their milk during the few first few weeks of their infants’ life and cannot feed them directly at the breast.

We defined three time points: (i) BMF was initiated in the NICU but discontinued prior to discharge (0–2 months); (ii) BMF continued after discharge but less than six months postpartum; (iii) BMF continued postpartum for six months or more.

Mothers were also asked about their pre-partum intentions to breastfeed as well as their reasons for discontinuation (when given). Reasons for discontinuing BMF were divided into three main groups: Maternal reasons: illness, hospitalization, end of maternity leave, new pregnancy, travel abroad, or a maternal wish to stop. Infant reasons: the infant’s medical condition did not allow BMF, the infant refused to feed directly at the breast, or the infant had difficulties latching and transitioning to solid food only. Failure of milk supply: Those who reported an inadequate supply of milk or a lack of milk altogether as their reason for discontinuation.

This study was approved by the SMC Institutional Review Board (project identification code: 3269-16-SMC; date of approval: 22 July 2016).

## 3. Statistical Analysis

Statistical analysis was conducted using SPSS software version 24 (SPSS, Chicago, IL, USA) for quantitative analysis. Differences between the study group and those lost to follow-up were assessed using a chi-square test for categorical variables and an independent sample *t*-test for continuous variables. Differences according to the three time points of BMF duration were assessed using a chi-square test for categorical variables and a one-way ANOVA for continuous variables. For the comparison between mothers who directly fed at the breast to those who expressed breast milk, we used a chi-square test for categorical variables and an independent sample *t*-test for continuous variables. Further, logistic regression analyses was applied to predict factors that may associate with BMF for ≥6 months and with feeding directly at the breast. A *p*-value less than 0.05 was considered statistically significant.

## 4. Results

**Figure 1:**
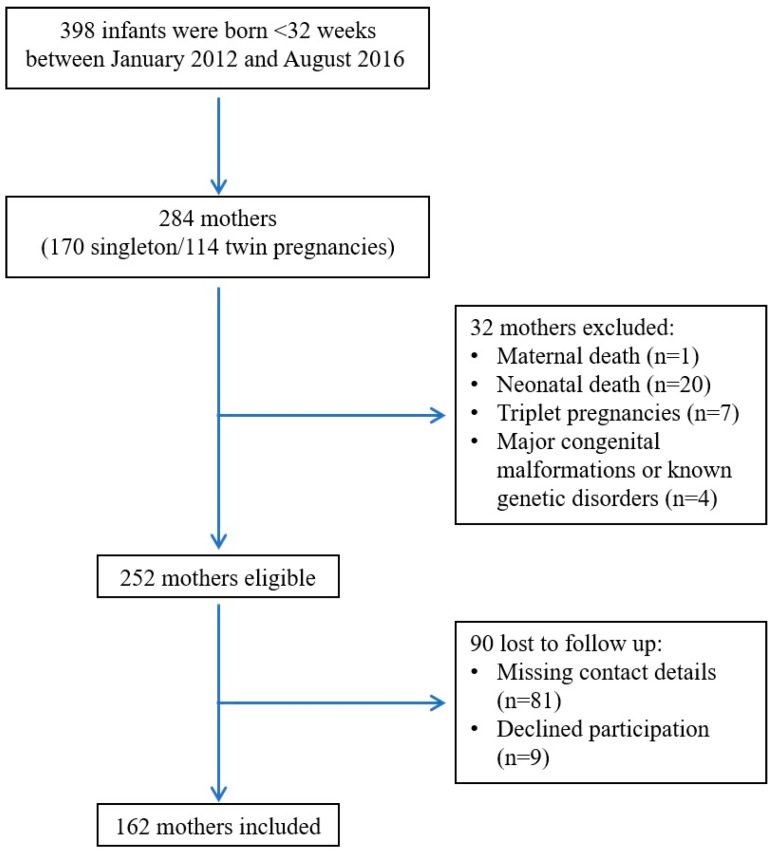
Flow chart of study cohort.

During the study period, 284 mothers gave birth to 398 infants born before 32 weeks of gestation. Thirty-two mothers (11.3%) were excluded for the following reasons: maternal death (*n* = 1), neonatal death (*n* = 20), triplets (*n* = 7), genetic abnormalities, or major congenital malformations (*n* = 4). Out of 252 eligible mothers (Figure 1), 162 (64.2%) replied to the questionnaire. Of the 90 mothers (35.7%) that did not respond, 81 could not be reached, and 9 declined to participate. Table 1 depicts the characteristics of the study group compared to those who were lost to follow-up. The groups were comparable in terms of maternal demographics and pregnancy characteristics.

Table 2 shows the maternal characteristics according to the three time points of BMF duration defined above. Of the 162 responders, 131 (80.8%) initiated BMF during NICU hospitalization. Of these, 22 (16.7%) discontinued BMF prior to discharge, 44 (33.5%) continued after discharge but discontinued before six months postpartum had elapsed, and 65 (49.6%) continued BMF for ≥6 months. Longer BMF (≥6 months) was significantly associated with direct feeding at the breast, longer duration EBMF, and singleton pregnancy. Intentions to breastfeed, previous experience and GA did not differ between the groups. Regression analysis adjusted to parity, singleton, and reasons for discontinuation of breast-feeding was conducted to predict the factors associated with BMF ≥ 6 months. Direct feeding at the breast (OR 5.5; 95% CI 2.00–15.37) and duration of EBMF (OR 1.5; 95% CI 1.25–1.88) emerged as the only significant predictors (Table 3). According to maternal reports, the main reason for earlier discontinuation of BMF (Time Points I and II) was inadequate supply of breast milk (61.9% and 63%, respectively). Infant causes were the most common reasons cited for late discontinuation (Time Point III) (49.2%), while inadequate breast milk supply was the least common reason (30%).

We then proceeded to explore predictors for direct feeding at the breast (any or only). Table 4 compares mothers who directly fed at the breast with mothers who exclusively expressed BM. The groups were comparable on most characteristics, including their initial intention to breastfeed. Mothers who directly fed at the breast were significantly more likely to have given birth to a singleton and were more likely to have longer duration of EBMF. We conducted a regression analysis to predict the factors associated with feeding directly at the breast adjusted to the number of infants (singleton or twin). Each month of EBMF was associated with a 1.1-fold increase in the chances of directly feeding at the breast (any or only) (95% CI 1.06–1.32) (Table 5). Causes for discontinuing BMF differed significantly between the groups, with 60.6% of the BMF mothers who exclusively expressed BM reporting a lack of breast milk supply compared to 28.3% of those who directly fed at the breast.

## 5. Discussion

The present study aimed to explore the effects of feeding mode (direct feeding at the breast vs. expressing breast milk), exclusivity (breast milk only vs. combined with formula), and maternal perceptions (intention to breastfeed and reasons for its discontinuation) on the duration of BMF among mothers of infants born at <32 weeks of gestation in a single medical center. The key findings of this study are that less than 50% of the mothers complied with World Health Organization (WHO) recommendations and continued BMF for six months or more. Direct feeding at the breast and the duration of EBMF were associated with longer BMF. Neither the intention to breastfeed nor previous experience was associated with its duration. An exploration of predictors for direct feeding at the breast showed that each month of EBMF was associated with a 1.2-fold increase in the chances of directly feeding at the breast. Mothers who discontinued earlier (<6 months) reported an inadequate supply of BM as the most common reason. These findings suggest that targeting direct feeding at the breast and EBMF may play a key role in prolonging BMF duration among preterm infants born at <32 weeks of gestation.

A gradual increase in BMF rates among preterm infants in NICUs has been reported [9,15]. Scientific evidence for the beneficial medical effects of BMF has been suggested as contributing to this change, as has implementation of intervention programs that promote BMF in the NICUs, such as those recommended by the Baby Friendly Hospital Initiative and by individualized developmental care [15,21,25,26,27,28,29,30,31,32,33]. In spite of the above, the rate of early weaning after discharge home is still high [19].

Direct feeding at the breast of very preterm infants is challenging for multiple reasons: Breast milk pumping for several weeks is required before the preterm infants are stable enough to attempt nutritive sucking at the breast [34,35]. Moreover, poor oral motor skills make the transition to direct feeding at the breast more challenging and stressful [36,37]. Consequently, maternal insecurity regarding adequate milk supply is high [7]. Little is known about how direct feeding at the breast of preterm infants in the NICU is related to BMF duration. In a study that included mothers of both term and preterm infants (27–36 weeks GA), breast milk expression rates were similar regardless of whether the infants were singletons or multiples. Feeding solely at the breast, particularly during the first two months postpartum, was associated with increased likelihood for longer BMF compared to breastmilk expression, regardless of whether combined with formula feeding or not [38]. Two small studies that focused on preterm infants support our finding by demonstrating a positive effect of direct feeding at the breast and BMF duration. Pindea at el. studied 66 preterm infants (24–35 weeks GA) whose mothers initiated BMF in the NICU. Factors that were positively associated with BMF at discharge included whether the infant was ever put to the breast (*p* < 0.0001) as well as the number of times the infant was put directly to the breast in the hospital (*p* < 0.0005) [39]. Briere et al. conducted a retrospective study that included 46 preterm infants (<32 weeks GA)) and found that infants who had ≥1 direct feedings at the breast per day in the NICU were more likely to be BMF one- and four-months post-discharge [40]. The results of the current study support the need for interventions to promote direct BMF among mothers of preterm infants. Such interventions may include increasing maternal awareness of the advantages of direct feeding at the breast and close guidance, especially during the critical time of transitioning to oral feeding. As in many other NICU units, in our NICU, BMF is strongly encouraged. The Israeli Ministry of Health set a target of >80% of feedings as breastmilk, offering a financial reward to NICUs that achieve this goal. Nevertheless, feeding mode was not set as a target. We suggest that promoting direct feeding at the breast among preterm infants could be a valuable intervention for prolonging BMF duration.

In our recently published study, mainly BMF (>75%) at Day 14 of life was associated with a longer duration of BMF [24]. We concluded that failure to engage in BMF exclusively at an early stage may be seen as a red flag for early BMF discontinuation. The results of the present study support the above conclusion by demonstrating a significant association between EBMF duration and BMF duration. In a study from Brazil that monitored 103 preterm infants, the median duration of BMF among preterm infants was five months (less than WHO recommendations). The risk of BMF interruption was three times higher among preterm infants who were receiving supplementation at the first outpatient visit than among those who were EBMF at the first consultation [41]. In general, EBMF is laborious, time-consuming and difficult and is marked by rather high discontinuance rates as time from delivery increases [16,42,43] or when maternity leave is short [43,44,45,46,47]. The results of the present study suggest that targeting prolonged BMF exclusivity may also help in achieving a longer duration of BMF among preterm infants.

A large Danish national cohort study that included 1747 infants born at <37 weeks (of them 317 born at <32 weeks) found that test-weighing the infants and minimizing pacifier use had a protective effect, while the use of nipple shields and delayed expressing of breast milk (>48 h) were associated with a negative effect on exclusive breastfeeding at discharge (defined as infant feeding directly at and from the breast but not from bottle or other device). Inadequate breastfeeding duration, which in that study was defined as four months plus half the amount of time the infant was born before estimated date of delivery, was more common when breast milk expression started <12 h after giving birth [22]. That study shares some similarities with the present study as both used questionnaires and investigated factors associated with BMF duration among preterm infants. The Danish study, however, was prospective and longitudinal, started soon after delivery, and continued until 12 months corrected age. Moreover, the Danish health system supports BMF by offering longer maternity leave (10.5 months), allowing longer duration of hospitalization (until exclusive BMF is well established or given up), and providing initial conditions that allow most mothers the opportunity to room-in in the NICU at least part of the time [48]. These different and unique baseline characteristics make the outcomes difficult to compare or to be generalized, as in many developed countries outside of Scandinavia mothers have significantly less supportive conditions. The studies also differ in the outcome measures (exclusive breast-feeding at discharge vs. any or exclusive BMF regardless of whether by bottle or direct), the study population (<37 weeks vs. <32 weeks) and the studied interventions (timing of expressing breast milk, use of pacifier or nipple shield, and test weighing in the Danish study vs. breast milk exclusivity, direct feeding at the breast, or expressing breast milk in the present study).

Eighty-two percent of the mothers who did not directly feed at the breast reported pre-partum intentions to do so. Among mothers who did not directly feed at the breast, BMF duration and exclusivity were significantly shorter (4.5 vs. 11.8 and 2.5 vs. 5.6 months, respectively) than for those who fed directly at the breast. Sixty percent of those who did not directly feed at the breast reported inadequate or a lack of milk supply as the main reason for BMF cessation. Previous studies have shown that maternal concerns regarding adequate milk supply are the main reason for discontinuing BMF [16,49,50]. These concerns may be heightened among mothers of preterm infants, especially during the transition from gavage feeding to direct feeding at the breast. Mothers experience their preterm infants as fragile and vulnerable and have more difficulties interpreting their infants’ hunger and satiety cues [51]. Moreover, NICU practices during the transition to direct feeding at the breast may increase a mother’s insecurity regarding her own milk supply. One study that included term infants showed a direct association between an infant’s weight and direct feeding at the breast four weeks postpartum. The authors proposed that health providers may have an impact on the duration of BMF by expressing concerns that infants with lower birth weights are not growing fast enough. Hence, mothers may subsequently be influenced to express their milk so they can carefully measure the amount produced and consumed [52]. In our NICU, mothers are commonly asked to weigh their babies before and after direct feeding at the breast (especially during transitioning) or to express breastmilk in order to assess its amount. These kinds of recommendations may increase insecurity as well as contribute to a loss of spontaneity. It is possible that BMF exclusivity and direct feeding at the breast are affected by maternal lack of confidence/insecurity in the ability to produce enough milk rather than by the actual absolute amount of milk produced. Nevertheless, this study was not designed to answer these questions.

The present study is not free of limitations. First, mothers were asked retrospectively about their pre-partum intentions to breastfeed and their BMF habits during the first year postpartum. This methodology is prone to inaccuracies, as has already been shown by Fleurant et al. In that study, mothers of very-low-birth-weight (VLBW) infants changed their human milk provision goals multiple times over the course of NICU hospitalization [53]. A second limitation is related to the definition of "direct feeding at the breast" that is either combined with bottle-feeding or exclusive (any or only). The superiority of one over the other has not been examined, but may be of importance. Combining direct feeding at the breast with expressed breast milk given by bottle may have a major impact on quality of life, as it gives mothers independence while maintaining an efficient and ongoing lactation process [13,54,55]. If this is the case, such a recommendation may be more easily adhered to. Further research regarding, this matter is warranted, as studies are inconsistent and the current study protocol is not suitable for answering this question.

## 6. Conclusions

In this cohort, only half of the mothers who initiated BMF in line with WHO recommendations continued with BMF ≥6 months. Our main findings suggest that (i) longer EBMF and direct feeding at the breast are directly associated with BMF duration; (ii) maternal perceptions regarding failure to produce enough milk and or to transfer it successfully to the infant are the most common reasons for early BMF discontinuation; (iii) early BMF discontinuation is more common among mothers who do not directly feed at the breast. These findings suggest that targeting direct feeding at the breast and EBMF may play a key role in prolonging the duration of BMF for preterm infants in the NICU.

Further studies are needed in order to assess interventions such as early skin to skin and breast milk expression, maternal rooming-in, minimizing use of pacifier, test-weighting, as well as consultant availability, during and after discharge, which would encourage mothers of preterm infant to engage in breast milk feeding exclusively and to feed directly at the breast.

## Figures and Tables

**Table 1:** Comparison between study group and those lost to follow-up.

	Study Group *n* = 162	Lost to Follow-Up *n* = 90	*p* *
Maternal age (years) M ± SD	31.8 ± 5.9	32.2 ± 6.1	0.632
In vitro fertilization	45 (27.8)	21 (23.6)	0.596
Twin pregnancy	63 (38.9)	39 (43.8)	0.265
Surgical delivery	105 (64.8)	59 (66.3)	0.463
Spouse	152 (93.8)	77 (90.6)	0.247
First child	76 (46.9)	38 (42.7)	0.306
Income level			
Low	51 (33.8)	30 (41.1)	
Middle	50 (33.1)	19 (26)	0.465
High	50 (33.1)	24 (32.9)	
Education ≤ 12 years	43 (27.6)	27 (37.5)	0.088
Gestational age	29.6 ± 1.9	29.7 ± 1.9	0.555
≤28	37 (22.8)	18 (20.3)	0.782
28.1–30	45 (27.8)	23 (25.8)	
30.1–32	80 (49.4)	48 (53.9)	

Data represented as number (%) or mean ± SD; * *p*-value is based on the results of an independent sample t test for continuous variables and a chi-square test for categorical one.

**Table 2:** Maternal characteristics according to the duration of breast milk feeding (BMF).

	BMF During Initial Hospitalization (Time Point I) *n* = 22	BMF Continued Post-Discharge but Discontinued < 6 Months (Time Point II) *n* = 44	BMF Discontinued ≥ 6 Months (Time Point III) *n* = 65	*p* *
Duration of BMF (month)	1.8 ± 0.3	3.9 ± 0.9	12.3 ± 7.0	<0.001
Duration of EBMF (month)	1.0 ± 0.9	1.9 ± 1.8	6.2 ± 6.1	<0.001
Directly feeding at the breast	1 (5.0)	12 (27.3)	45 (70.3)	<0.001
Planned to directly feed at the breast	17 (89.5)	36 (81.8)	55 (85.9)	0.707
Previous experience with BMF	6 (31.6)	13 (29.5)	25 (39.7)	0.527
Maternal age (years)	32.3 ± 5.5	32.3 ± 5.9	31.2 ± 5.6	0.534
In vitro fertilization	8 (36.4)	12 (27.3)	12 (18.5)	0.527
Twin pregnancy	14 (63.6)	14 (31.8)	21 (32.3)	0.021
Surgical delivery	11 (50)	31 (70.5)	40 (61.5)	0.262
Spouse	20 (90.9)	42 (95.5)	62 (95.4)	0.693
Income level				
Low	8 (40)	11 (28.2)	19 (30.6)	0.548
Middle	8 (40)	14 (35.9)	18 (29.0)	0.548
High	4 (20)	14 (35.9)	25 (40.3)	
Education ≤ 12 years	7 (35)	10 (23.8)	13 (20.3)	0.404
First child	7 (31.8)	21 (47.7)	32 (49.2)	0.349
Gestational age (weeks)	30.3 ± 1.8	29.4 ± 2.1	29.4 ± 1.9	0.161
≤28	4 (18.2)	12 (27.3)	17 (26.2)	
28.1–30	3 (13.6)	12 (27.3)	19 (29.2)	0.388
30–32	15 (68.2)	20 (45.5)	29 (44.6)	
Reason for discontinuation ^1^				
Maternal	6 (27.3)	16 (36.4)	25 (38.5)	0.637
Infant	4 (18.2)	3 (6.8)	32 (49.2)	<0.001
Failure to supply	13 (61.9)	28 (63.6)	18 (30.0)	0.001

Data represented as number (%) or mean ± SD; missing values were less than 9%; BMF = breast milk feeding; EBMF = exclusive breast milk feeding; * *p*-value is based on the results of one-way ANOVA test for continuous variables and a chi-square test for categorical one. ^1^ overlapping which allow >100%.

**Table 3:** Results of logistic regression analysis predicting BMF > 6 months.

	OR	95% CI	*p* *
Direct feeding at the breast	5.54	2.00–15.37	0.001
Duration of EBMF	1.53	1.25–1.88	<0.001
Twin pregnancy	1.27	0.47–3.41	0.641
Reasons for discontinuation of breast-feeding			
Child	0.33	0.09–1.27	0.106
Failure to supply	1.19	0.40–3.60	0.747

BMF = breast milk feeding; EBMF = exclusive breast milk feeding; OR = Odds ratio; CI = confidence interval; * *p*-value is based on the results of logistic regression analysis.

**Table 4:** Comparison between mothers who fed directly at the breast (any or only) vs. those who exclusively expressed breast milk.

	Directly Feeding at the Breast (Any or Only) *n* = 58	Expressed Breast Milk *n* = 71	p *
Duration of BMF (month)	11.8 ± 7.9	4.5 ± 2.8	<0.001
Duration of EBMF (month)	5.6 ± 6.5	2.5 ± 2.7	<0.001
Planned to feed directly at the breast	50 (87.7)	57 (82.6)	0.294
BMF of previous child	24 (42.9)	20 (28.6)	0.069
Reason for discontinuation ^1^			
Maternal	23 (39.7)	23 (32.4)	0.0251
Infant	31(53.4)	7 (9.9)	<0.001
Failure to supply	15 (28.3)	43 (60.6)	<0.001
In vitro fertilization	11 (19.0)	22 (31.0)	0.122
Maternal age	31.4 ± 5.4	32.1 ± 6.0	0.439
≤25	6 (10.3)	9 (12.7)	0.808
25–34	36 (62.1)	38 (53.5)	0.808
35–39	11 (19.0)	16 (22.5)	
≥40	5 (8.6)	8 (11.3)	
Twin pregnancy	17 (29.3)	32 (45.1)	0.049
Spouse	56 (96.6)	67 (94.4)	0.440
Income level			
Low	13 (24.1)	23 (35.4)	0.248
Middle	17 (31.5)	22 (33.8)	0.248
High	24 (44.4)	20 (30.8)	
Gestational age (weeks)	29.8 ± 1.7	29.4 ± 2.1	0.294
≤28	11 (19.0)	21 (29.6)	0.347
28.1–30	17 (29.3)	16 (22.5)	0.347
>30	30 (51.7)	34 (47.9)	
Education ≤ 12 years	12 (21.4)	17 (25.0)	0.401
First child	26 (44.8)	33 (46.5)	0.496
Surgical delivery	45 (63.4)	64 (66.7)	0.390

Data represented as number (%) or mean ± SD; missing values were less than 9%; ^1^ overlapping which allow >100%; BMF = breast milk feeding; EBMF = exclusive breast milk feeding. * *p*-value is based on the results of an independent sample *t*-test for continuous variables and a chi-square test for categorical ones.

**Table 5:** Results of logistic regression analysis predicting feeding directly at the breast.

	OR	95% CI	*p* *
Duration of EBMF (month)	1.18	1.06–1.32	0.002
Twin pregnancy	0.60	0.28–1.30	0.195

EBMF = exclusive breast milk feeding; OR = Odds ratio; CI = confidence interval; * *p*-value is based on the results of logistic regression analysis.

## Supplementary Materials

The following are available online at www.mdpi.com/2072-6643/9/11/1202/s1, Study questioner.

## Availability of Data and Materials

The datasets used and/or analyzed in this study are available from the corresponding author on reasonable request.

## Abbreviations

AAP	American Association of Pediatrics
BM	Breast milk
BMF	Breast milk feeding
EBMF	Exclusive breast milk feeding
GA	Gestational age
NICU	Neonatal intensive care unit
WHO	World Health Organization
