# Supplementary material for: Direct Feeding at the Breast Is Associated with Breast Milk Feeding Duration among Preterm Infants
Source: Nutrients. 2017 Nov 1;9(11):1202. doi: 10.3390/nu9111202 (PMC5707674; doi:10.3390/nu9111202)
Supplement: Supplementary file 1 [file nutrients-09-01202-s001.pdf]

<u>Data collection questionnaire on breastmilk feeding among mothers who gave birth to preterm infants (born before 32 weeks of GA) during 2012-2015</u>

Questionnaire for participating mothers

Mother's first and last name________

Child's name ___________________

Child's date of birth ________________

Date of completing the questionnaire ____________________

- Was the pregnancy a twin pregnancy? Yes / No
- If the answer is yes, was this baby the first or the second twin? 1st / 2nd
- Was your baby fed by breastmilk (in any way: directly at the breast, pumped breastmilk)? Yes/No

<u>If your baby was fed breastmilk:</u>

- How long was your baby fed **exclusively** by breastmilk (**exclusively** = without any additional infant formula, breast milk only)? ___________
- How long was your baby fed by breastmilk (even combined with formula)? _____________________
- How did your baby receive the breastmilk (directly at the breast only (nursing) /pumped breastmilk only/both)?
- Why did you discontinue feeding your baby breastmilk? (e.g., end of maternity leave and need to go back to work/ inadequate supply of milk / personal preference / other) _________________

<u>If your baby was **not** fed breastmilk:</u>

- What was the reason for not feeding your baby with breastmilk? _______________________
- During your pregnancy, before you gave birth prematurely, did you intend to breastfeed your baby? Yes/No
- Did you nurse (directly breastfeed) previous children? Yes/No
- Did you pump breastmilk with previous children? Yes/No
- If so, what problems did you encounter? ______________________
  What helped you? ______________________________________

Thank you for completing the questionnaire.
If you have any further questions please feel free to ask Dr. IM, Dr. LL, or Dr. SP
